# Regulation of oxidative stress resistance in *Campylobacter jejuni*, a microaerophilic foodborne pathogen

**DOI:** 10.3389/fmicb.2015.00751

**Published:** 2015-07-29

**Authors:** Jong-Chul Kim, Euna Oh, Jinyong Kim, Byeonghwa Jeon

**Affiliations:** School of Public Health, University of Alberta, EdmontonAB, Canada

**Keywords:** *Campylobacter jejuni*, oxidative stress, stress response, regulation of gene expression, survival mechanisms

## Abstract

*Campylobacter jejuni* is one of the leading bacterial causes of human gastroenteritis. Due to the increasing rates of human campylobacteriosis, *C. jejuni* is considered as a serious public health concern worldwide. *C. jejuni* is a microaerophilic, fastidious bacterium. *C. jejuni* must overcome a wide range of stress conditions during foodborne transmission to humans, such as food preservation and processing conditions, and even in infection of the gastrointestinal tracts of humans. Particularly, this microaerophilic foodborne pathogen must survive in the atmospheric conditions prior to the initiation of infection. *C. jejuni* possesses unique regulatory mechanisms for oxidative stress resistance. Lacking OxyR and SoxRS that are highly conserved in other Gram-negative foodborne pathogens, *C. jejuni* modulates the expression of genes involved in oxidative stress resistance mainly via the peroxide resistance regulator and *Campylobacter* oxidative stress regulator. Based on recent findings of ours and others, in this review, we described how *C. jejuni* regulates the expression of oxidative stress defense.

Food safety is a serious public health issue in both developed and developing countries. Consumption of food contaminated with pathogens results in gastrointestinal diseases, chronic sequelae and disability, and even death (Lindsay, 1997). *Campylobacter jejuni* is considered as the most common bacterial cause of acute gastroenteritis in humans (Park, 2002), and develops clinical symptoms, such as diarrhea, abdominal cramps, and fever (Altekruse et al., 1999). Also, *C. jejuni* is the primary cause of Guillan–Barré syndrome, an acute peripheral neuropathy (Hughes and Cornblath, 2005).

The Centers for Disease Control and Prevention (CDC) estimated that *Campylobacter* annually causes approximately 2.4–4 million infection cases in the US, and campylobacteriosis is the second major foodborne infection in the country (Samuel et al., 2004). In Canada, *Campylobacter* is the most frequent bacterial cause of acute gastrointestinal illness, accounting for approximately 0.6 million infection cases per year (Thomas et al., 2006). Worldwide, the estimated number of annual cases of human campylobacteriosis reaches up to 400–500 million (Ruiz-Palacios, 2007). Despite the enormous number of infection cases, *Campylobacter* infection has not been controlled, but rather exhibits a still increasing trend (CDC, 2014). In addition, the resistance of *Campylobacter* to clinically important antibiotics, such as fluoroquinolones, has emerged as another serious public health concern (Luangtongkum et al., 2009). In some countries, antibiotic resistance is highly prevalent in *C. jejuni*, seriously compromising the effectiveness of antimicrobial chemotherapy. For example, ciprofloxacin (an important fluoroquinolone antibiotic for humans) resistance is found in approximately 92% *C. jejuni* isolates from raw chicken in South Korea (Han et al., 2007), and 100% *C. jejuni* isolates from children in Thailand (Serichantalergs et al., 2007).

## *Campylobacter* Biology and Transmission

*Campylobacter* sp. are isolated from a wide range of animals, such as poultry, cattle, sheep, and dogs, and most of them are associated with various disease symptoms in animals and humans (Humphrey et al., 2007). As a microaerophilic bacterium, *Campylobacter* requires an oxygen concentration of 3–15%, and cannot ferment nor oxidize carbohydrates; instead, amino acids are utilized as the major energy source (Brenner and Staley, 2005). While campylobacters grow at 35–37°C, some species, such as *C. jejuni, C. coli, C. lari, C. helveticus*, and *C. hyointestinalis*, are thermotolerant (Brenner and Staley, 2005). As a pathogenic species that is most commonly implicated in human infection (Scharff, 2012), *C. jejuni* is frequently isolated from poultry, possibly because the body temperature of poultry is close to the optimal growth temperature of *C. jejuni* (Young et al., 2007), which is 42°C (Hazeleger et al., 1998; Stintzi, 2003). Although *C. jejuni* is a thermotolerant species with an optimal growth temperature higher than that of *Escherichia coli* and *Salmonella, C. jejuni* is more sensitive to heat stress than these bacteria. The *D*-value of *C. jejuni* is 2.12 min at 55°C in ground chicken meat (Blankenship and Craven, 1982), whereas the *D*-values of *E. coli* O157:H7 and *Salmonella* in chicken are 8.76–9.74 min at 55°C and 3.2 min at 56°C, respectively (Ahmed et al., 1995; Mazzotta, 2000).

Due to the commensalism of *C. jejuni* in the intestinal tracts of various animals, consumption of animal products is the leading cause of human infections with *C. jejuni*. Since poultry is the major reservoir for *C. jejuni*, this pathogenic bacterium is likely to be present in poultry wastes with an average population level of ca. 10^5^ CFU/g in fecal samples collected from broiler chicken flocks (Chen and Jiang, 2014). In particular, high levels of *Campylobacter* contamination in poultry meat can lead to extensive cross contamination in food processing (Jorgensen et al., 2002; Chen and Jiang, 2014). Raw meat products from other food-producing animals, such as beef, pork, and lamb, have also been implicated in the transmission of *Campylobacter* (Kramer et al., 2000). Whereas sporadic *Campylobacter* infections are frequently associated with poultry, outbreaks are often related to dairy products (Taylor et al., 2013). Unpasteurized milk is considered as a potential vehicle transmitting *C. jejuni* to humans (Hudson et al., 1999; Frost et al., 2002), because cattle is the second major reservoir for *C. jejuni* (Jonas et al., 2015). Also, many outbreak cases report that water may serve as an important environmental reservoir for *Campylobacter* (Bolton et al., 1982; Thomas et al., 1999), because animal feces and contaminated soil are associated with *Campylobacter* contamination of water (Thomas et al., 1999). Interestingly, thermotolerant *Campylobacter* sp., such as *C. jejuni, C. coli*, and *C. lari*, have also been isolated from seafood and shellfish (Wilson and Moore, 1996; Endtz et al., 1997; Frost et al., 2002), and some human campylobacteriosis cases have been attributed to the consumption of raw shellfish (Endtz et al., 1997; Frost et al., 2002). Since *C. jejuni* is prevalent in various animals and environmental niches, this fastidious microaerophilic bacterium will be exposed to a myriad of stress conditions in both animal hosts and external environments (Murphy et al., 2006; Jackson et al., 2009). More than any other stress conditions, especially, increased oxygen tension in the atmosphere will be the most viability-threatening stress that *C. jejuni* cannot avoid encountering during transmission. In this review, we described current information available for the genetic/protein elements of oxidative stress resistance in *C. jejuni* as well as their functions and regulation.

## *Campylobacter jejuni*’s Defense Against Oxidative Stress

### What is Oxidative Stress?

The oxygen concentration in the atmosphere plays a crucial role in bacterial growth in the environment. Generally, bacteria possess redox enzymes that mediate oxidative phosphorylation with oxygen molecule as an electron carrier, and the use of oxygen molecule inevitably generates reactive oxygen species (ROS), such as the superoxide anion (O^2-^) and hydrogen peroxide (H_2_O_2_). Subsequently, intracellular ferrous iron (Fe^2+^) and H_2_O_2_ produce a highly reactive hydroxyl radical (HO⋅) via the Fenton reaction (Storz and Imlay, 1999). If not detoxified, ROS gives deleterious effects on macromolecules in bacteria, such as DNA, membranes, and proteins (Imlay, 2003). Although bacteria possess mechanisms to maintain the homeostasis of ROS, the alteration in oxygen concentrations affects the generation rate of ROS, which leads to oxidative stress (Lushchak, 2011). Despite *C. jejuni*’s capabilities to harmonize oxidative stress under aerobic and microaerobic conditions by utilizing various electron donors and acceptors (Jeon et al., 2010), the survival of *C. jejuni* is directly subject to the oxygen concentrations of its surrounding environments. According to a DNA microarray study, exposure to aerobic conditions affects biological process and gene expression in *C. jejuni*; particularly, the expression of genes associated with oxidative phosphorylation, antioxidation, and nucleic acid metabolism is increased by the oxygen concentration (ca. 21% O_2_) in the atmosphere (Kaakoush et al., 2009). Described below is a list of genes/proteins that thus far have been reported to affect oxidative stress in *C. jejuni* directly or indirectly.

### Superoxide Stress Defense

Superoxide dismutase (SOD) is the primary enzyme that is responsible for the detoxification of superoxide (Winterbourn et al., 1975). Whereas *E. coli* possesses three *sod* genes including *sodA, sodB*, and *sodC*, which encode manganese-, iron-, and copper, zinc-cofactored SOD, respectively (Imlay, 2008), *C. jejuni* expresses only the iron-cofactored SodB (Pesci et al., 1994), and a *sodB* mutation increases *C. jejuni* susceptibility to both superoxide (e.g., menadione) and peroxide stress (e.g., H_2_O_2_ and cumene hydroperoxide) (Palyada et al., 2009; Flint et al., 2014). According to a two dimensional gel electrophoresis analysis (2DGE), paraquat (i.e., superoxide) exposure overexpresses Cj1371, a putative periplasmic protein, which is homologous to VacJ (virulence-associated chromosome locus J) in *Shigella flexneri*, and a muation of Cj1371 increases *C. jejuni* susceptibility to paraquat (Suzuki et al., 1994; Garénaux et al., 2008a). In *S. flexneri*, VacJ contibutes to the maintenance of lipid asymmetry in the outer membrane, intercellular spread, and resistance to sodium dodecyl sulfate (SDS; Carpenter et al., 2014); however, its molecular function has not been defined in *C. jejuni*. Bacterial motility also indirectly contributes to *C. jejuni* resistance to superoxide stress, as mutations of genes involved in flagellar biosynthesis and modification (e.g., *motAB, flgR, flhB, flgD*, and *pseB*) render *C. jejuni* significantly sensitive to menadione, a superoxide generator, and slightly to H_2_O_2_ (Flint et al., 2014). It would be because flagellar mutations disrupt the proton potential for flagellar rotation and the electron leakage increases the generation of ROS, including the superoxide anion, and affects oxidative stress in *C. jejuni* (Flint et al., 2014).

### Peroxide Stress Defense

Catalase, which decomposes H_2_O_2_ to H_2_O and O_2_, plays an important role in oxidative defense mechanism (Imlay, 2008). The *katA* gene is the sole catalase gene present in *C. jejuni*. Studies using a *katA* knockout mutant have revealed the primary role of *katA* in scavenging H_2_O_2_ under oxidative stress and in the survival of *C. jejuni* within macrophages (Grant and Park, 1995; Day et al., 2000). The heme-trafficking protein Cj1386, which is located downstream of *katA*, is involved in the full catalase activity and contributes to *C. jejuni* colonization of chicken intestines (Flint et al., 2012). *C. jejuni* has three different antioxidant enzymes in the peroxiredoxin family, including alkyl hydroperoxide reductase (AhpC), and two putative peroxidases [i.e., thiol peroxidase (Tpx) and bacterioferritin comigratory protein (Bcp)] (Baillon et al., 1999; Parkhill et al., 2000). In *Salmonella enterica*, alkyl hydroperoxide reductase consists of two subunit AhpC and AhpF; the former is a peroxide reducing part and the latter is a flavoprotein that uses NAD(P)H as an electron donor to transfer electrons to AhpC (Jacobson et al., 1989; Storz et al., 1989). Unlike *Salmonella* and *E. coli, C. jejuni* possesses only *ahpC* and lacks an *ahpF* homolog in the genome (Baillon et al., 1999). AhpC expression is controlled by iron concentrations at the transcriptional level, and iron restriction increases the AhpC transcription level (Baillon et al., 1999). An *ahpC* knockout mutant is highly sensitive to cumene hydroperoxide (an organic peroxide), but not to H_2_O_2_ (Baillon et al., 1999), possibly because *C. jejuni* AhpC would scavenge endogenous H_2_O_2_ at physiological concentrations as *E. coli* AhpC does (Seaver and Imlay, 2001). Two Tpxs (i.e., Tpx and Bcp) are commonly involved in H_2_O_2_ defense, and Bcp reduces also organic peroxides, such as cumene hydroperoxide and *tert-*butyl hydroperoxide (Atack et al., 2008). Cytochrome *c* peroxidase (CCP) is a periplasmic protein that reduces H_2_O_2_ to water. *C. jejuni* possesses two putative CCP genes; *docA* and *Cjj0382* (Parkhill et al., 2000; Hendrixson and DiRita, 2004). Even though the two genes exhibit the common characteristics of CCP, such as heme-binding periplasmic proteins, none of them contribute to H_2_O_2_ resistance in *C. jejuni* 81–176 (Bingham-Ramos and Hendrixson, 2008). In *C. jejuni* NCTC 11168, however, a mutation of *Cj0358*, which is *Cjj0382* in *C. jejuni* 81–176, is associated with H_2_O_2_ resistance (Flint et al., 2014), suggesting that *Cj0358* (*Cjj0382*) would affect oxidative stress defense strain-dependently. Also, the autoinducer 2-depdendent quorum sensing affects oxidative stress depending on the strain. A *luxS* mutant in *C. jejuni* NCTC 11168 exhibits comparable resistance to oxidative stress to the wild type (Elvers and Park, 2002); however, a *luxS* mutation in *C. jejuni* 81–176 increases the sensitivity to peroxides (H_2_O_2_ and cumene hydroperoxide; He et al., 2008). The rubredoxin oxidoreductase/rubrerythrin chimeric protein Rrc is a non-heme iron protein, interacts with exogenous and endogenous H_2_O_2_, and confers resistance to menadione (i.e., superoxide) and H_2_O_2_ (Yamasaki et al., 2004; Flint et al., 2014).

Complementary mechanisms have been reported to protect *C. jejuni* from oxidative stress by scavenging free intracellular irons associated with ROS generation or by repairing cellular damages caused by ROS. The *E. coli* Dps (DNA binding protein from starved cells) is known to protect DNA by sequestrating free Fe^2+^ and by reducing the formation of ROS (Zhao et al., 2002). In *C. jejuni*, similarly, Dps captures free Fe^2+^ and confers resistance to H_2_O_2_ (Ishikawa et al., 2003). Upon activation by Fe^2+^ or H_2_O_2_, Dps binds to and protects DNA from damages from hydroxyl radicals (Huergo et al., 2013). Methionine sulphoxide reductases (MsrA and MsrB) reduces oxidized methionine (i.e., methionine sulphoxide [Met-SO]) to methionine and restores methionine function in protein synthesis (Ezraty et al., 2005). Mutations of *msrA* and *msrB*, especially an *msrA*/*B* double mutation, sensitize *C. jejuni* to peroxide and superoxide stress and nitrosative stress as well (Atack and Kelly, 2008). CmeG is a multidrug eﬄux pump that belongs to the major facilitator superfamily (MFS), playing a role in antimicrobial resistance in *C. jejuni*. Particularly, CmeG overexpression significantly increases *C. jejuni* resistance to fluoroquinolone antibiotics. In addition to the role in antibiotic resistance, interestingly, CmeG significantly affects *C. jejuni* susceptibility to H_2_O_2_ (Jeon et al., 2011).

Although a number of genes and proteins have been reported to contribute to oxidative stress defense in *C. jejuni* (**Table 1**), a recent extensive mutagenesis and colonization study done by Flint et al. (2014) revealed that *ahpC, katA*, and *sodB* play the primary role in the oxidative stress defense of *C. jejuni*.

**Table 1:** Proteins associated with oxidative stress defense in *C. jejuni.*

Name	Functions	Susceptibility increase by mutation	Reference
Superoxide dismutase (SodB)	Dismutation of superoxide	Superoxide (MND)^∗^ Organic peroxide (CHP)^∗∗^ H_2_O_2_	Palyada et al. (2009), Flint et al. (2014)
Cj1371	Homolog of *Shigella flexneri* VacJ	Superoxide (PQ)^∗∗∗^	Garénaux et al. (2008a)
Methionine sulphoxide reductases (MsrA/B)	Repair of oxidized methionine	H_2_O_2_ Organic peroxide (CHP) Superoxide (PQ) Nitrosative stress	Atack and Kelly (2008)
Rubredoxin oxidoreductase /rubrerythrin-like protein (Rrc)	Unknown	H_2_O_2_ Superoxide (MND)	Yamasaki et al. (2004), Flint et al. (2014)
Catalase	Decomposition of H_2_O_2_	H_2_O_2_	Grant and Park (1995)
Cj1386	Heme trafficking to KatA	H_2_O_2_	Flint et al. (2012)
Alkyl hydroperoxide reductase (AhpC)	Reduction of alkyl peroxides Scavenger of endogenous H_2_O_2_	Organic peroxide (CHP)	Baillon et al. (1999)
Thiol peroxidase (Tpx) and Bacterioferritin comigratory protein (Bcp)	Scavenger of H_2_O_2_ Reduction of peroxides	In a *tpx/bcp* double mutant H_2_O_2_ Organic peroxide (CHP) Superoxide (PQ) Nitrosative stress	Atack et al. (2008)
Cytochrome *c* peroxidases (CCPs)	Reduction of H_2_O_2_	None in *C. jejuni* 81-176 H_2_O_2_ in a *Cj0358* mutant of *C. jejuni* NCTC 11168	Bingham-Ramos and Hendrixson (2008), Flint et al. (2014)
S-ribosylhomocysteinase (LuxS)	Synthesis of autoinducer-2	None in *C. jejuni* NCTC 11168 H_2_O_2_ and organic peroxide (CHP) in *C. jejuni* 81–176	Elvers and Park (2002), He et al. (2008)
DNA binding protein from starved cells (Dps)	Sequestration of free Fe^2+^ Protection of DNA from oxidative damage	H_2_O_2_	Ishikawa et al. (2003), Huergo et al. (2013)
CmeG, a multidrug eﬄux transporter	Excretion of toxic compounds	H_2_O_2_	Jeon et al. (2011)

*^∗^MND, menadione; ^∗∗^CHP, cumene hydroperoxide; ^∗∗∗^PQ, paraquat.*

## Oxidative Stress and *C. jejuni*’s Stress Response

Bacteria coordinate complex regulatory systems to achieve effective response to different stress conditions by removing the sources of stress and by repairing cellular damages resulting from the stress (Kültz, 2005; Guo and Gross, 2014). Many different stress situations activate general stress response to cross-protect bacteria from various stress conditions; “cross-protection” means that cells exposed to one stress also become resistant to other stress (Hengge-Aronis, 2002; Capozzi et al., 2009; Wesche et al., 2009). In addition to the primary role of ROS detoxification, oxidative stress defense is also associated with other stress response mechanisms. In *E. coli*, oxidative stress is involved in bacterial response to a variety of stress conditions, such as heat, salinity, heavy metals, and lactic acid bacteria (Imlay, 2015). Oxidative stress is associated even with antimicrobial killing of pathogens (Kohanski et al., 2007). Treatment of *E. coli* and *S. aureus* with bactericidal antibiotics stimulates bacterial respiration, resulting in the depletion of NADH and the generation of toxic ROS (Kohanski et al., 2007). The implication of oxidative stress in antimicrobial lethality is under debate (Imlay, 2015), because antimicrobial activity still persists in the absence of oxygen (Liu and Imlay, 2013) and this antimicrobial mechanism appears to be influenced by unique bacterial metabolisms, particularly tricarboxylic acid pathway (Feld et al., 2012). Nevertheless, antimicrobial killing of *C. jejuni* is affected by oxidative stress. Mutations of *katA* and *sodB* affect the bacterial lethality of ciprofloxacin and rifampicin with a reduction in the minimal inhibitory concentrations (MICs) by about twofold (Hwang et al., 2013). As discussed below, oxidative stress also affects *C. jejuni* response to different stress conditions that this foodborne pathogen may encounter during its transmission to humans via food.

### Temperature Stress

Most foodborne pathogens are originated from the gastrointestinal tracts of animals and usually grow optimally at temperatures close to the body temperatures of host animals. Foodborne pathogens may experience dramatic temperature changes during food processing, storage, and preparation (Capozzi et al., 2009), and resist the stress of temperature changes by producing heat- and cold-shock proteins (Becker and Craig, 1994; Wouters et al., 2000; Beales, 2004). Interestingly, oxidative stress is related to *C. jejuni* response to temperature stress. The catalase activity increases in *C. jejuni* in proportion to temperatures (Hazeleger et al., 1998), but *C. jejuni* is more susceptible to oxidative stress at high temperature (i.e., 42°C) than low temperatures (i.e., 4°C), suggesting that temperature affects oxidative stress resistance in *C. jejuni* (Garénaux et al., 2008b). Exposure to cold-shock increases the expression of *sodB* and *Cj0358* (a CCP; Stintzi and Whitworth, 2003), and a *sodB* mutation makes *C. jejuni* and *C. coli* more susceptible to freeze-thaw stress (Stead and Park, 2000; Garénaux et al., 2009), suggesting that oxidative stress influences *C. jejuni* ability to survive under freeze-thaw conditions.

### Acid Stress

Since low pH conditions are frequently adopted to food preservation, foodborne pathogens may encounter acid stress in foods (Hill et al., 1995). In addition, passage through the stomach would be the most demanding challenge for foodborne pathogens during infection (Chowdhury et al., 1996). In *E. coli* O157:H7, acid exposure alters the transcriptional level of oxidative stress genes, including *oxyR* and *soxS*, which are key regulators of oxidative stress defense (Allen et al., 2008). Dps is an iron-sequestration ferritin protein and contributes to both acid and oxidative stress defense by protecting *E. coli* O157:H7 from DNA damage (Choi et al., 2000; Jeong et al., 2008). In *C. jejuni*, similarly, the expression of oxidative stress defense genes, such as *dps, sodB, trxB*, and *ahpC*, is increased by exposure to HCl or acetic acid (Birk et al., 2012). Pre-exposure of *C. jejuni* to aerobic culture increases bacterial survival under acid stress, suggesting that oxidative stress is linked to acid stress response (Murphy et al., 2003).

### Osmotic Stress

The use of osmolytes as food preservatives decreases water activity and contributes to the control of microbial growth in food (Beales, 2004). Under high osmotic pressure, cells activate defense systems to prevent shrinkage and plasmolysis (Beales, 2004; Chung et al., 2006). In *Bacillus cereus*, pre-exposure to 1% NaCl increases resistance to H_2_O_2_ (Browne and Dowds, 2001). Exposure of *B. cereus* to both mild (2.5% NaCl) and severe (5% NaCl) salt stress also increases expression of oxidative stress defense genes, such as *ahpC, katA*, and *katE*, along with genes associated with iron homeostatis, such as ferrochelatase (den Besten et al., 2009). Similarly, exposure of *C. jejuni* to 1% NaCl up-regulates oxidative stress genes, such as *katA* and *sodB*, over an extended exposure time from 15 min to 6 h, suggesting oxidative stress defense is involved in osmotic stress response (Cameron et al., 2012).

### Nutrient Starvation

Nutrient starvation in foodborne pathogens can occur at any time during transmission to humans, such as growth on the surface of food processing equipment. Starvation stress induces cross-protection against heat, oxidative, and osmotic stress in *E. coli* (Jenkins et al., 1988, 1990). *Enterococcus faecalis* develops a multi-resistance state against heat, H_2_O_2_, acid, and ethanol after exposure to carbohydrate-limited environments (Giard et al., 1996). In stationary phase (starvation-like condition), *C. jejuni* undergoes a physiological switch from acetate production to acetate uptake, and the oxidative stress genes, such as *perR, ahpC, sodB*, and *tpx*, are up-regulated during the metabolic switch (Wright et al., 2009).

As described above, oxidative stress is associated with *C. jejuni*’s response to various stresses; however, it remains unexplained how oxidative stress is related to different stress conditions in *C. jejuni*. Similar to the mechanism for antibiotic killings via oxidative stress, presumably, exposure to stress may affect metabolisms and electron transport systems, consequently resulting in ROS generation. Stress conditions may compromise *C. jejuni*’s capability to maintain the homeostasis of ROS, and *C. jejuni* would further require the function of oxidative stress defense systems to cope with the instability in ROS homeostasis under the stress conditions.

## Effect of Oxidative Stress on Survival Mechanisms in *C. jejuni*

As a common stress response mechanism, oxidative stress defense also plays a role in the survival mechanisms of *C. jejuni*, such as aerotolerance, biofilm formation, and induction of a viable-but-non-culturable (VBNC) state. Biofilm formation is one of extensively studied survival mechanisms in pathogenic bacteria in stress environments (Parsek and Singh, 2003). Biofilm formation on food processing equipment is a major concern to food industry, since biofilms may persistently release microorganisms and act as a source of microbial contamination (Hall-Stoodley and Stoodley, 2005). Bacteria in biofilms are physiologically different from cells in a planktonic state and highly resistant to chemical disinfectants and antibiotics (Fux et al., 2005). When residing in biofilms, *C. jejuni* easily acquires antibiotic resistance genes because DNA is a major structural material of biofilms and *C. jejuni* is naturally competent in DNA uptake (Bae et al., 2014). Oxidative stress is associated with biofilm formation in foodborne pathogens. According to a proteomic analysis of the protein expression profiles in *E. coli* O157:H7 biofilms, the protein expression levels of Tpx and SodC increase in biofilms, and *tpx*, and *sodC* mutants exhibit a significant defect in biofilm formation (Kim et al., 2006). Similarly, the expression of oxidative stress resistance proteins AhpC and Tpx also increases in *C. jejuni* biofilms (Kalmokoff et al., 2006). In *C. jejuni*, inactivation of *ahpC* increases the accumulation of the total ROS and lipid peroxides, and significantly enhances biofilm formation (Oh and Jeon, 2014). Antioxidant treatment reduces the enhanced biofilm formation in the *ahpC* mutant to the wild-type level (Oh and Jeon, 2014), suggesting that oxidative stress is one of signals that induce biofilm formation in *C. jejuni*. *C. jejuni* is microaerophilic; however, *C. jejuni* forms biofilms more vigorously in oxygen-rich (i.e., aerobic) conditions than in oxygen-limited (i.e., microaerobic) conditions (Reuter et al., 2010). This would be because increased oxidative stress under aerobic conditions may enhance biofilm formation. We also observed that antioxidant treatment decreased the levels of biofilm formation under aerobic conditions (unpublished data).

Oxidative stress also impacts the entry of *C. jejuni* into a VBNC state. Bacteria in a VBNC state are alive but not culturable by traditional microbiological methods, and removal of inducing stress resuscitates VBNC cells and allows them to grow in laboratory conditions (Oliver, 2010). VBNC cells are characterized by the typical size reduction and coccoid cellular morphology (Oliver, 2010). The length of an individual *C. jejuni* cell is 6 μm; however, the average length of *C. jejuni* VBNC cell is approximately 1.19 μm (Thomas et al., 2002). Several factors have been reported to induce a VBNC state in *C. jejuni*, including as temperature, starvation, formic acid, and aerobic conditions (Kassem et al., 2013). Our recent study reported significant morphological changes in *C. jejuni* from rod-spiral to coccoid forms after exposure to oxygen-rich conditions (Kim et al., 2015), and our further characterization demonstrated that increased oxidative stress induces the formation of VBNC *C. jejuni* under aerobic conditions (Oh et al., 2015). Oxidative stress also affects the induction of a VBNC state in *Vibrio vulnificus*. An *oxyR* mutant of *V. vulnificus* that is defective in catalase activity easily enters a VBNC state compared to the parent strain (Kong et al., 2004). Supplementation of H_2_O_2_-degrading enzyme and ROS scavenging compounds, such as catalase and sodium pyruvate, enhances resuscitation of *E. coli* O157 and *V. vulnificus* from a non-culturable state (Mizunoe et al., 1999; Bogosian et al., 2000), indicating alleviation of oxidative stress promotes bacterial resuscitation from a VBNC state.

Aerotolerance would be the survival mechanism that is most closely connected to oxidative stress. Although obligate anaerobes and microaerohiles reside in habitats under oxygen-limited conditions to avoid oxidative stress, the oxygen-sensitive bacteria still possess conserved oxidative stress resistance systems to survive in oxygen-rich conditions (Chiang and Schellhorn, 2012). Several studies have demonstrated that genes of oxidative stress defense contribute to aerotolerance in *C. jejuni*. Mutations of *ahpC* and its upstream gene *fdxA* significantly decreases aerotolerance in *C. jejuni* (Baillon et al., 1999; van Vliet et al., 2001), and two thiol peroxidases, Tpx and Bcp, contribute to *C. jejuni*’s aerotolerance (Atack et al., 2008). More ROS accumulates under aerobic conditions than microaerobic conditions with increased oxidation of proteins and lipids (Oh et al., 2015). Although *katA, sodB*, and *ahpC* are key enzymes of ROS detoxification, *ahpC* plays a more important role in *C. jejuni* survival under oxygen-rich conditions than *katA* and *sodB* (Oh et al., 2015).

## Regulation of Oxidative Stress Defense

### Oxidative Stress Defense Regulators in Other Bacteria

Oxidative stress defense is controlled by complicated regulatory systems. Most well-characterized regulatory systems of oxidative stress defense would be SoxRS and OxyR, which are dedicated to the regulation of superoxide and peroxide defense, respectively, in *E. coli* and *Salmonella* (Imlay, 2008). SoxR (superoxide response regulator) was first identified as a genetic locus that positively regulates protein expression after exposure to superoxide-generating agents, such as paraquat (Greenberg et al., 1990; Tsaneva and Weiss, 1990). Redox-cycling drugs generating the superoxide anion directly activate SoxR (Gu and Imlay, 2011), and the activated SoxR stimulates the expression of SoxS, which subsequently induces oxidative stress defense genes (Pomposiello et al., 2001). OxyR is activated by H_2_O_2_ through the oxidation of two cysteine residues (i.e., Cys 199 and Cys 208) and the formation of a disulfide bond (Zheng et al., 1998; Imlay, 2008). Oxidized OxyR binds to promoters co-operatively with the RNA polymerase in *E. coli* (Tao et al., 1993), and positively regulates a group of peroxide stress defense genes, such as *ahpCF, dps*, and *katG*, whose expression is also induced by H_2_O_2_ (Zheng et al., 2001). PerR (peroxide resistance regulator) is the major regulator of peroxide stress defense in Gram-positive bacteria, such as *Bacillus subtilis, Staphylococcus aureus*, and *Streptococcus pyogenes*, and in some Gram-negative bacteria, including *C. jejuni* and *Helicobacter hepaticus* (van Vliet et al., 1999; Mongkolsuk and Helmann, 2002; Belzer et al., 2011). As a member of Fur (ferric uptake regulator) family of metallo-regulators, *B. subtilis* PerR senses the intracellular Fe/Mn ratio and requires metal ions, including Zn^2+^ as a structural ion, and Mn^2+^ and Fe^2+^ as regulatory ions (Helmann, 2014). While Fe^2+^ mediates PerR regulation of peroxide defense genes, such as *katA, ahpCF*, and *mrgA* (a homolog of *dps*), the negative auto-regulation of *perR* involves Mn^2+^ in *B. subtilis* (Fuangthong et al., 2002). Although *B. subtilis* PerR regulates peroxide defense genes, *perR* transcription is not affected by H_2_O_2_ (Fuangthong et al., 2002). Instead, conformational changes in the PerR protein by H_2_O_2_ stress contribute to the regulatory function of PerR. Oxidation of one of two histidine residues (i.e., H37 and H91) by H_2_O_2_ in *B. subtilis* PerR results in the dissociation of Fe^2+^ from PerR, and the demetallated PerR cannot bind to DNA, and this conformational changes in PerR induce gene expression (Lee and Helmann, 2006).

### Oxidative Stress Defense Regulators in *C. jejuni*

**FIGURE 1:**
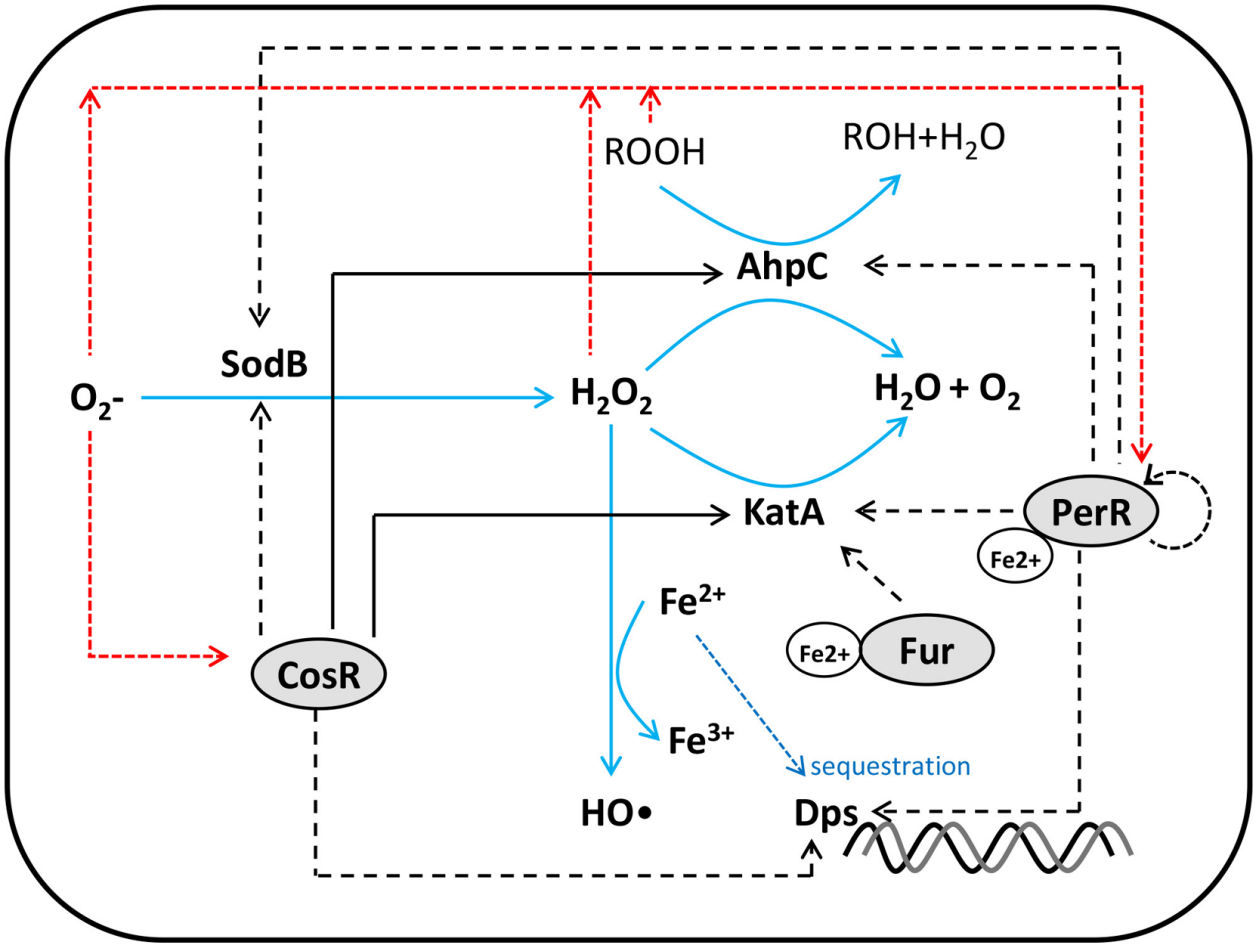
**Schematic diagram for *Campylobacter* oxidative stress regulator (CosR) (Hwang et al., 2011b, 2012), ferric uptake regulator (Fur) (Butcher et al., 2012), and peroxide resistance regulator (PerR) (van Vliet et al., 1999; Palyada et al., 2009; Kim et al., 2011, 2015) regulation of oxidative stress resistance in *C. jejuni*.** Positive and negative regulations are indicated by black solid and dotted lines, respectively. Red dotted lines show transcriptional or translational down regulation by reactive oxygen species (ROS).

Unlike other Gram-negative foodborne pathogenic bacteria, such as *E. coli* and *Salmonella enterica*, the *soxRS* and *oxyR* homologs are not found in the *C. jejuni* genome (Parkhill et al., 2000). Instead, PerR and CosR have been characterized relatively well in the regulation of oxidative stress defense in *C. jejuni* (**Figure 1**).

(1) *PerR*: The *C. jejuni* genome contains two genes encoding Fur homologs. Whereas Fur regulates iron uptake genes (van Vliet et al., 1998; Palyada et al., 2004; Holmes et al., 2005), another Fur homolog, PerR, is involved in the regulation of peroxide stress resistance (van Vliet et al., 1999). As PerR is rarely present in Gram-negative bacteria, *C. jejuni* is the first Gram-negative bacterium that has been shown to possess PerR (van Vliet et al., 1999). Similar to the regulatory patterns of *B. subtilis* PerR (Bsat et al., 1998), a *perR* mutation significantly increases the expression of antioxidant proteins, KatA and AhpC, rendering the *C. jejuni perR* mutant hyper-resistant to peroxides, such as H_2_O_2_ and cumene hydroperoxide (van Vliet et al., 1999). A microarray analysis done by Palyada et al. (2009) further demonstrated that PerR directly or indirectly regulates at least 104 genes, including those of oxidative stress defense and motility. Although *C. jejuni* PerR shares 32% identity to *B. subtilis* PerR (Kim et al., 2011) and shows functional similarities in peroxide defense regulation, *C. jejuni* PerR exhibits some unique differences from *B. subtilis* PerR. The transcription of *perR* is driven by two consecutive, overlapping promoters, and the PerR box overlaps with the entire -35 box of the upstream promoter and a part of the downstream promoter (Kim et al., 2011). Similar to *B. subtilis, perR* transcription is negatively auto-regulated in *C. jejuni*; thus, PerR binding to the *perR* promoter interferes with *perR* transcription (Kim et al., 2011). Whereas *perR* auto-regulation involves manganese in *B. subtilis* (Fuangthong et al., 2002), *perR* autoregulation in *C. jejuni* is mediated by iron, not by manganese (Kim et al., 2011).

Recently, we showed oxidative stress defense plays a role in *C. jejuni* survival under aerobic conditions (Oh et al., 2015). Aerobic culture of *C. jejuni* significantly increases the transcriptional levels of *ahpC, katA*, and *sodB* by ROS accumulation, and a *perR* mutation abrogated the transcriptional induction of the three antioxidant genes by oxidants, demonstrating PerR modulates transcriptional changes in response to increased oxidative stress under aerobic conditions in *C. jejuni* (Kim et al., 2015). Interestingly, PerR binds to the *sodB* promoter and negatively regulates the transcription of *sodB*, the most representative gene in superoxide defense (Kim et al., 2015). These findings suggest that PerR regulates both peroxide and superoxide defense in *C. jejuni*. The possibility of PerR regulation of *sodB* has also been suggested in a review article of van Vliet et al. (2002) based on a sequence analysis by using a putative PerR binding sequence in Gram-positive bacteria in spite of the issue in sequence similarities between PerR and Fur boxes. Unlike *E. coli* and *Salmonella* that regulate oxidative stress defense with stress-specific regulators, such as OxyR and SoxRS, *C. jejuni* PerR regulates the transcription of both peroxide and superoxide defense genes.

As forementioned, PerR regulation of oxidative stress defense genes in *B. subtilis* is mediated by conformational changes in the PerR protein in response to oxidative stress (Lee and Helmann, 2006). The same mechanism is likely to be present in *C. jejuni*, because two histidine residues (i.e., H37 and H91) are conserved in *C. jejuni* PerR (Kim et al., 2011). In addition, PerR regulation of oxidative stress is also mediated at the *perR* transcriptional level; both peroxide and superoxide reduce the level of *perR* transcription regardless of the presence and absence of iron (Kim et al., 2015). Taken together, exposure to oxygen-rich conditions, such as aerobic conditions, increases the intracellular ROS levels in *C. jejuni*, which subsequently decreases *perR* transcription, resulting in the derepression of oxidative stress defense genes.

(2) *Fur*: Fur is a key transcriptional regulator in the control of iron homeostasis in *C. jejuni* (van Vliet et al., 1998). In addition, its contribution to oxidative stress regulation has been suggested in *C. jejuni*. Catalase activity increases by a *perR* mutation twofold less in iron-rich conditions than iron-limited conditions, and a *perR/fur* double mutation results in comparable levels of catalase activities irrespective of iron, meaning that PerR and Fur co-regulates peroxide resistance (van Vliet et al., 1999). A recent chromatin immunoprecipitation and microarray analysis of Butcher et al. (2012) shows that holo-Fur (i.e., iron-bound Fur) represses *katA* and activates *rrc*, whereas apo-Fur represses *rrc*. Similarities in the binding sites of PerR and Fur have been an issue in the determination of the regulon of each regulator (van Vliet et al., 2002). In fact, the PerR box is more similar to the holo-Fur box for repression than the Fur box for activation in *C. jejuni* based on two recent studies (Kim et al., 2011; Butcher et al., 2012). Given the repression of *katA* by both PerR and holo-Fur and their binding site similarities, it is still not clear how the two iron-associated transcriptional regulators coordinate *katA* expression in *C. jejuni*. Nevertheless, PerR appears to play a more important role than Fur, because a *perR* mutation results in more significant increase in catalase activity and H_2_O_2_ resistance than a *fur* mutation does (van Vliet et al., 1998).

(3) *CosR*: CosR (*Campylobacter* oxidative stress regulator) is an OmpR-type essential response regulator that plays an important role in the control of oxidative stress resistance in *C. jejuni* (Hwang et al., 2011b, 2012). The *C. jejuni* genome contains six histidine kinases, 11 response regulators, and an hybrid of histidine kinase and response regulator (CheA; Parkhill et al., 2000; Marchant et al., 2002). In an extensive mutagenesis study, Raphael et al. (2005) endeavored to knock out 11 putative two-component response regulators, but *cosR* (*Cj0355c*), and *cprR* (*Cj1227c*) mutants could not be generated, suggesting that these two genes are essential in *C. jejuni*. Garénaux et al. (2008a) reported that exposure of *C. jejuni* to paraquat, a superoxide generator, reduces the protein level of CosR (Cj0355c) in a 2DGE analysis, suggesting a potential role of CosR in oxidative stress defense. In the two studies above, the essentiality of *cosR* was an obstacle to functional characterization of CosR, because it is not possible to generate its knock-out mutant. Thus, the function of CosR has been characterized in an alternative way by using antisense-mediated gene knockdown (Hwang et al., 2011b), which was technically established in *C. jejuni* first by Jeon and Zhang (2009). A 2DGE analysis of protein expression profiles under CosR knockdown and overexpression conditions identified 32 proteins whose expression is significantly affected by CosR, revealing CosR regulates positively AhpC, and negatively SodB, Dps, and Rrc (Hwang et al., 2011b). A further characterization of CosR regulon by using a DNA microarray exhibited CosR positively regulates *katA* expression, and CosR knockdown and overexpression affect catalase activity (Hwang et al., 2012). CosR regulation of oxidative stress defense genes (i.e., *ahpC, dps, katA, sodB*) is direct based on the results of electrophoretic mobility shift assays and DNase I footprinting assays (Hwang et al., 2011b, 2012). Additionally, CosR negatively regulates the *cmeABC* operon encoding the major multidrug eﬄux pump CmeABC in *C. jejuni*, and several genes of bacterial motility, such as *flgD, flgE, flgL*, and *fliK*, suggesting the pleiotropic regulation of CosR in *C. jejuni* (Hwang et al., 2012). The CosR protein level is reduced by paraquat, but not by H_2_O_2_ (Hwang et al., 2011b), indicating that CosR specifically senses superoxide stress, although CosR regulates both peroxide and superoxide resistance genes. Since superoxide is the first line of toxic by-product from oxygen reduction cycle, superoxide sensing would probably be more efficient than peroxide sensing in *C. jejuni* response to oxidative stress. CosR also negatively regulates expression of LuxS (Hwang et al., 2011b), which affects oxidative stress resistance in a strain-dependent manner (see, Peroxide Stress Defense section above).

*Campylobacter* oxidative stress regulator homologs are predominantly found in bacteria that belong to ε-*Proteobacteria*, such as *Campylobacter, Helicobacter, Arcobacter*, and *Wolinella* (Hwang et al., 2011b). CosR appears to be an orphan response regulator, because a potential sensor kinase does not exist nearby the *cosR* gene in *C. jejuni* (Hwang et al., 2011b) as well as in other thermotolerant *Campylobacter* sp., such as *C. coli* and *C. lari* (Hwang et al., 2014). Interestingly, however, the sensor kinase CosS is present in non-theremotolerant *Campylobacter* sp., such as *C. fetus, C. concisus, C. curvus*, and *C. hominis*, and also in other members of ε-*Proteobacteria*, including *Wolinella* and *Arcobacter* (Hwang et al., 2014). Based on the inability to obtain a *cosR* knockout mutant in *C. fetus, cosR* appears to be essential in *C. fetus* (Hwang et al., 2014). A mutation of the sensor kinase *cosS* slightly reduces aerotolerance in *C. fetus*, but does not affect oxidative stress resistance (Hwang et al., 2014). Despite high similarities in the amino acid sequence in CosR homologs in thermotolerant and non-thermotolerant *Campylobacter* sp., the histidine kinase CosS in non-thermotolerant *C. fetus* does not mediate phosphotransfer to *C. jejuni* CosR, suggesting that CosR functions differentially between therotolerant and non-thermontolerant *Campylobacter* species (Hwang et al., 2014).

(4) *Other regulators*: In addition to the regulators mentioned above, several other regulators have been reported to affect oxidative stress defense in *Campylobacter*. The CprRS two-component system consists of an essential response regulator (CprR) and the sensor kinase CprS (Svensson et al., 2009). Due to the indispensability of CprR in *C. jejuni* survival, the *cprR* gene cannot be knocked out, but a *cprS* mutation, which is not lethal to *C. jejuni*, results in decrease in SodB, Rrc, LuxS, and CosR expression and increase in AhpC and KatA expression in a 2DGE analysis, also slightly increasing sensitivity to both peroxide and superoxide stress (Svensson et al., 2009). Further characterization of *cprS* revealed that CprRS contributes to the regulation of genes associated with bacterial envelope, such as *htrA, peb4, lepP, lspA*, and *gne* (Svensson et al., 2015). Cj1556 is a MarR family transcriptional regulator and positively regulates peroxide stress defense genes, such as *perR, katA*, and *ahpC*, whereas *sodB* is negatively regulated by Cj1556 (Gundogdu et al., 2011). A *Cj1556* mutation reduces *C. jejuni* capability to resist oxidative, aerobic, and heat stress and to survive in human intestinal epithelial cells and murine macrophages (Gundogdu et al., 2011). Cj1103 is an ortholog of the *E. coli* global posttranscriptional regulator CsrA (carbon starvation regulator) and functionally complements a *csrA* mutation in *E. coli* in the regulation of biofilm formation and motility (Fields and Thompson, 2012). Inactivation of *csrA* increases sensitivity to hydrogen peroxide and aerobic stress (Fields and Thompson, 2008). *C. jejuni* harbors only three sigma factors, including RpoD (σ^70^), RpoN (σ^54^), and FliA (σ^28^), and lacks stress-related sigma factors found in other bacteria (e.g., RpoS; Parkhill et al., 2000). RpoD and FliA are sigma factors that are dedicated to the transcription of housekeeping and flagella biosynthesis genes, respectively, and RpoN is known to be involved in flagella biosynthesis and bacterial motility in *C. jejuni* (Hendrixson et al., 2001). Although RpoN is not a regulator, RpoN has been shown to affect oxidative stress response in *C. jejuni* (Hwang et al., 2011a).

In summary, oxidative stress resistance affects: (1) bacterial response to various stress conditions that *C. jejuni* may encounter during foodborne transmission to humans, and (2) critical survival mechanisms, such as aerotolerance, biofilm and VBNC formation. *C. jejuni*’s resistance to and survival in stress conditions that are associated with oxidative stress defense may eventually increase the possibility of *C. jejuni* transmission to humans via food, resulting in food safety problems. Additionally, studies thus far have demonstrated that oxidative stress response in *C. jejuni* is controlled by many different regulatory mechanisms that are distinctly different from mechanisms reported in other foodborne pathogens. Investigation of molecular mechanisms for oxidative stress resistance in *C. jejuni* has been greatly facilitated by the availability of improved experimental techniques. Further characterization of the mechanisms for oxidative stress resistance and regulation will tell us how this fastidious microaerophilic pathogen survives in stress conditions and causes such huge problems in food safety.

## Conflict of Interest Statement

The authors declare that the research was conducted in the absence of any commercial or financial relationships that could be construed as a potential conflict of interest.
